# Alcohol Consumption During the COVID‐19 Pandemic: A Critical Review

**DOI:** 10.1002/hup.70004

**Published:** 2025-03-25

**Authors:** A. Merlo, P.A. Hendriksen, N.R. Severeijns, J. Garssen, G. Bruce, J.C. Verster

**Affiliations:** ^1^ Division of Pharmacology Utrecht Institute for Pharmaceutical Sciences Utrecht University Utrecht the Netherlands; ^2^ Danone Global Research & Innovation Center Utrecht the Netherlands; ^3^ Division of Psychology and Social Work School of Education and Social Sciences University of the West of Scotland Paisley UK; ^4^ Centre for Mental Health and Brain Sciences Swinburne University Melbourne Australia; ^5^ Cognitive Neurophysiology Department of Child and Adolescent Psychiatry Faculty of Medicine TU Dresden Dresden Germany

**Keywords:** alcohol consumption, COVID‐19, lockdown, pandemic

## Abstract

**Objective:**

The purpose of this systematic review was to summarize the impact of the 2019 coronavirus disease (COVID‐19) pandemic on individuals' alcohol consumption.

**Methods:**

PubMed was searched to identify relevant studies. Articles were included if they provided information on overall (changes in) alcohol consumption, and factors that may influence alcohol consumption including demographics, socioeconomic status, educational background, living situation, and health status. Following screening, 100 articles were identified and included in this review.

**Results:**

Overall findings show no change (51%) or a reduction (23%) in alcohol consumption during the COVID‐19 pandemic. However, across countries, on average 1 in 4 individuals reported an increase in alcohol consumption (26%), in particular during the COVID‐19 lockdown periods. Most common correlates of increased alcohol consumption were being female, having a child at home, higher educational level, and poorer mental health (including higher scores for stress, anxiety and depression).

**Conclusion:**

Although overall alcohol consumption was reduced during the COVID‐19 pandemic, a considerable subpopulation of drinkers increased their alcohol consumption.

## 1 Introduction

The 2019 Coronavirus disease (COVID‐19), which first emerged in Wuhan, China in December 2019, profoundly affected society and the economy on an unprecedented scale. The World Health Organisation (WHO) declared COVID‐19 a global pandemic on the 11th March 2020 (WHO 2020, 2022). In response, governments worldwide implemented restrictions commonly referred to as lockdowns, advising or mandating people to indoors and work remotely where possible. These restrictions varied between countries but generally included wearing facemasks, washing hands, and social distancing. In addition, various establishments such as restaurants, bars, gyms, and schools, were closed (Hirsch 2020; Brown et al. 2022). However, off‐premises alcohol outlets were allowed to remain open in most countries as they were considered essential shops, while other business adapted to the pandemic via selling alcoholic beverages through delivery services.

While COVID‐19 restrictions were designed to reduce virus transmission, they also had negative impacts on individuals' well‐being and mental health. Isolation and reduced face‐to‐face interactions led to mental health deterioration among individuals and families. Rising unemployment rates further contributed to financial hardships, impacting the well‐being of both the individual and their loved ones (Brooks et al. 2020; Fitzgerald et al. 2020). Additionally, among some individuals, the distressing experience of lockdown led to a decline in physical activity, an increase in sedentary behaviors, changes in food consumption, and an emergence of emotional eating behaviors (e.g., binge and comfort eating). These unhealthy lifestyle changes are concerning, as poor eating habits have been linked to depressive symptoms and anxiety (Ammar et al. 2020; Son et al. 2020; Catucci et al. 2021; Bühlmeier et al. 2022).

In the educational sector, the shift to online learning led to increased stress and, in some cases, poorer academic performance (Hendriksen et al. 2021). A study of college students in the United States found reduced concentration levels and lower self‐confidence, increased stress, and a decline in overall mental health (Son et al. 2020). Moreover, distancing measures augmented the deteriorated their mental health status. Furthermore, 86% of students reported worsened sleep patterns, which are known to be correlated with depressive symptoms and anxiety (Son et al. 2020). Other studies reported the use of negative coping mechanisms, including ignoring news relating to COVID‐19, spending more time sleeping, maladaptive eating patterns, and substance abuse (Fitzgerald et al. 2020; Son et al. 2020; Bakaloudi et al. 2022), including increased alcohol consumption (Tran et al. 2020; Killgore et al. 2021).

Alcohol use is often used as a coping strategy due to its short‐term effects of improving mood, decreasing anxiety, and alleviating stress and depression (Hasking et al. 2011). Previous research has shown an increase in alcohol consumption following significant life stressors (Flory et al. 2009; Chodkiewicz et al. 2020). Thus, increases in alcohol consumption during the COVID‐19 pandemic are not surprising. Unfortunately, frequent and high‐dose alcohol use can lead to negative health and well‐being issues (Rodriguez et al. 2020) including impaired cognitive abilities and motor skills, increased injury risk (Batra et al. 2016), and various diseases, including digestive, cardiovascular, and infectious diseases (WHO 2018; NIAAA 2023).

Of great importance with respect to COVID‐19 is the negative effect of alcohol consumption on the immune system (A. White and Hingson 2013). Alcohol can alter cytokine production and lead to changes in T‐ and B‐lymphocytes, natural killer cells and monocytes, which are used to fight off infection (Rocco et al. 2014). This alteration impairs the body's ability to combat unfamiliar diseases. During a global pandemic, it is crucial to maintain a strong and healthy immune system. In this context, Merlo, Severeijns, et al. (2021) proposed a model demonstrating the relationship between alcohol consumption, immune fitness, and the presence and severity of COVID‐19 symptoms. Taken together, it is essential to consider the effects of excessive alcohol consumption on the immune system and susceptibility to COVID‐19, and factors influencing alcohol consumption.

This review aimed to summarize the current literature on COVID‐19 and alcohol consumption, examining changes across different populations during the lockdown. Factors include sex, age, race, educational level, mental health status, employment status, and living situation, relationship status, income, student status, binge drinking, proximity to or risk of COVID‐19, and social support.

## 2 Methods

**FIGURE 1:**
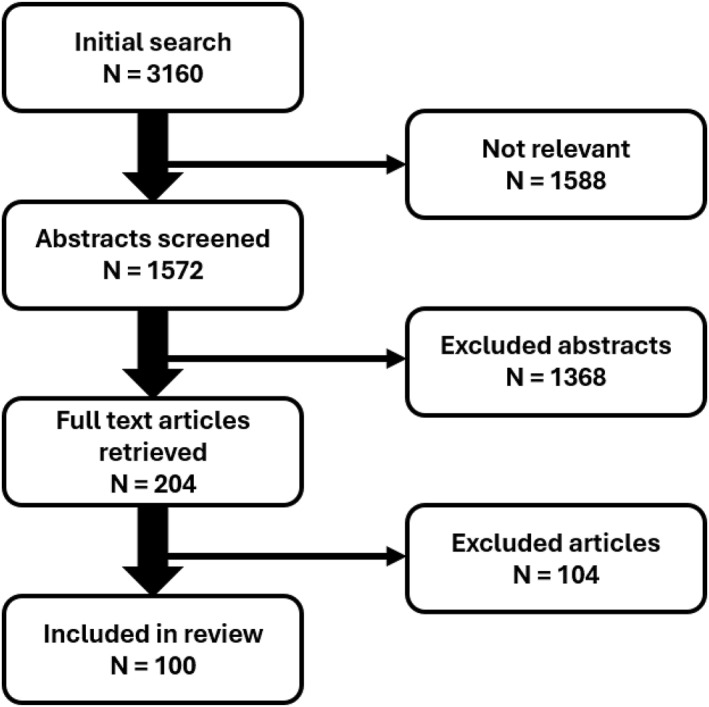
Flow chart of the literature search.

A systematic literature search was conducted (15 January 2024) using the online database PubMed (See Figure 1). Search terms used included “COVID‐19” and “alcohol use.” English language articles during the period of January 2020 and December 2023 were included, yielding 3160 hits. Inclusion criteria comprised those articles concerning alcohol consumption patterns during the COVID‐19 pandemic. Full‐text articles were examined and those not relevant were discarded, including those that did not report at least one of the following topics: living alone or together, sex, age, race, income, students versus non‐students, differences in educational level, relationship status, mental health status, presence of children at home/living situation, employment/work status, working in healthcare, binge drinking, social support, harmful drinking, presence or risk of COVID‐19. Articles investigating alcohol consumption patterns during the COVID‐19 pandemic of participants with an underlying disease were excluded. One reviewer independently searched and identified relevant articles based on the above criteria (A.M.). Following this, a second reviewer (P.H.) checked the search and confirmed the eligibility of the studies. Consequently, 100 articles were selected. Studies included both cross‐sectional and longitudinal designs. Most studies comprised self‐reported web‐based surveys, with measures of alcohol consumption differing across studies, ranging from quantity to frequency, patterns such as binge drinking, number of drinks on the heaviest drinking occasion, and the Alcohol Use Disorders Identification Test (AUDIT). The studies were conducted in various countries, such as the U.S., UK, Argentina, Australia, Belgium, Poland, Slovakia, Greece, Norway, Spain, China, France, Canada, as well as multinational studies.

## 3 Findings

The reviewed studies suggest significant variability in alcohol consumption patterns during COVID‐19 pandemic (See Table 1). Overall, most studies found that respondents reported not changing their drinking behavior during lockdown (51% across studies) (see Table 1). However, of the other half, some reported reducing their alcohol intake (23% across studies), while about the same percentage of respondents reported an increase of their alcohol consumption during the COVID‐19 pandemic (26% across studies).

**TABLE 1:** Percentage change of alcohol consumption.

	Country	Decreased	No changes	Increased
Bloomfield et al. (2022)	Denmark	39%	34%	27%
Coulaud et al. (2022)	Canada	44%	33%	23%
Coulaud et al. (2022)	France	45%	21%	34%
Koyun et al. (2022)	Germany	43%	36%	21%
Panagiotidis et al. (2020)	Greece	44%	32%	21%
Rodriguez et al. (2020)	Spain	58%	32%	10%
Sallie et al. (2020)	UK	45%	19%	36%
Yan et al. (2020)	China	39%	35%	26%
Acuff et al. (2022)	Cross country	23%	54%	23%
Avery et al. (2020)	USA	11%	75%	14%
Biddle et al. (2020)	Australia	27%	53%	20%
Bramness et al. (2021)	Norway	30%	57%	13%
Bühlmeier et al. (2022)	Germany	20%	57%	23%
Capasso et al. (2021)	USA	20%	51%	29%
Chodkiewicz et al. (2020)	Poland	18%	68%	14%
Clare et al. (2021)	Australia	24%	57%	19%
Garnett et al. (2021)	UK	26%	48%	26%
Gerritsen et al. (2021)	New Zealand	9%	58%	33%
Górnicka et al. (2020)	Poland	11%	71%	18%
Håkansson (2020)	Sweden	10%	82%	8%
Hanafi et al. (2021)	Indonesia	30%	45%	25%
Hendriksen et al. (2022)	Argentina	33%	56%	12%
Ingram et al. (2020)	UK	26%	39%	35%
Jaffe et al. (2021)	USA	17%	43%	40%
Kilian et al. (2021)	Europe	30%	50%	20%
Knell et al. (2020)	USA	11%	50%	39%
Koopmann et al. (2020)	Germany	19%	38%	35%
Kriaucioniene et al. (2020)	Lithuania	16%	70%	14%
Merlo, Hendriksen, et al. (2021)	Netherlands	26%	50%	24%
Mongeau‐Pérusse et al. (2021)	Canada	4%	71%	25%
Moura et al. (2023)	Brazil	33%	44%	23%
Oksanen et al. (2021)	Finland	27%	48%	25%
Oldham et al. (2021)	UK	20%	50%	30%
Robinson et al. (2020)	UK	31%	41%	28%
Rodríguez‐Pérez et al. (2020)	France	14%	68%	18%
Rolland et al. (2020)	France	12%	68%	18%
Rossinot et al. (2020)	France	20%	62%	18%
Sanchez et al. (2020)	USA	10%	64%	26%
Scarmozzino and Visioli (2020)	Italy	10%	53%	37%
Schmits and Glowacz (2022)	Belgium	25%	49%	26%
Sharma et al. (2020)	USA	9%	67%	24%
Sidor and Rzymski (2020)	Poland	0%	85%	15%
Somé et al. (2022)	Canada	13%	53%	34%
Stanton et al. (2020)	Australia	23%	55%	22%
Steffen et al. (2021)	Germany	25%	60%	15%
Szajnoga et al. (2021)	Poland	39%	43%	18%
Tran et al. (2020)	Australia	12%	67%	21%
Valente et al. (2021)	Latin America	33%	53%	14%
Vanderbruggen et al. (2020)	Belgium	14%	56%	30%
Weerakoon et al. (2021)	USA	13%	45%	42%
Zajacova et al. (2020)	Canada	10%	76%	14%
Grossman et al. (2020)	USA	13%	27%	60%
Salerno et al. (2021)	USA	32%	27%	41%
Sen et al. (2021)	Indonesia	26%	21%	53%
Silczuk (2020)	Poland	9%	38%	53%
**Average**	**All countries**	**23%**	**51%**	**26%**

*Note:* The bold values indicate the average percentages and gray shaded values indicate the highest percentages.

Several factors could explain altered drinking behavior during the COVID‐19 pandemic. Restrictions on social events and gatherings, and the closure of alcohol selling establishments are some examples of contributory factors to individuals' reduction in alcohol consumption (Biddle et al. 2020; Stanton et al. 2020). Additionally, individual and sample differences play a role. For instance, among students, moving back home with parents following campus closure was protective against heavy drinking resulting in an overall decrease in alcohol consumption (H. R. White et al. 2020; Merlo, Hendriksen, et al. 2021; Ryerson et al. 2021).

In contrast, an increase in alcohol use was reported by an average of 26% of the population. Reasons for this include “treating oneself” and “fewer consequences of drinking more” (Biddle et al. 2020). Other correlates include demographic differences, employment and relationship status, and childcare responsibilities. A U.S. study of 1540 participants showed a 14% increase in alcohol consumption during the pandemic, with an additional drink per day reported by most. This increase was more pronounced among females (17%) and 30‐ to 60‐year‐olds (19%) (Salerno et al. 2021). Similar trends were observed in Europe, with studies in Belgium, Poland, the UK, and Greece reporting greater alcohol consumption during the pandemic (Grossman et al. 2020; Panagiotidis et al. 2020; Vanderbruggen et al. 2020; Kilian et al. 2021). Thus, various factors, including changes in daily routines, psychological states, and demographic differences, can all impact drinking behaviors. The following sections will explore these factors in more detail.

### 3.1 Demographic Factors

#### 3.1.1 Sex Differences

While a trend of females being more likely to have changed their drinking behaviors emerged among the retrieved articles, a cross‐sectional study looking at drinking frequency, the quantity of alcohol consumed, and the incidence of heavy episodic drinking events in 21 European countries shows no sex differences (Kilian et al. 2021). These findings are further supported by other papers reporting no significant differences between females and males (Chodkiewicz et al. 2020; Grossman et al. 2020; Neill et al. 2020; Robinson et al. 2020; Zajacova et al. 2020; Schmits and Glowacz 2022). One study showed evidence of significant altered drinking patterns for younger females. According to this study among Norwegian participants, younger females were more likely to report a change in alcohol use compared to men, with both decreasing and increased drinking (Bramness et al. 2021).

Additionally, an American study outlined that females may be more prone to develop risky drinking behaviors during social distancing periods, hypothesizing that females are generally more likely to be involved in and responsible for childcare. Combined with working from home and home schooling, additional stressors might explain the increased alcohol consumption among females (Pollard et al. 2020). Other studies also reported a higher quantity and/or frequency of alcohol use among females (Boschuetz et al. 2020; Pollard et al. 2020; Rodriguez et al. 2020; Silczuk 2020; Barbosa et al. 2021; Capasso et al. 2021; Salerno et al. 2021; Steffen et al. 2021; Wozniak et al. 2021; Acuff et al. 2022). Fewer studies claimed observing greater alcohol consumption among males (Gritsenko et al. 2021; Killgore et al. 2021; Valente et al. 2021) or a decrease in consumption among females (Callinan et al. 2021; Steffen et al. 2021).

#### 3.1.2 Age

A trend emerged for young to middle‐aged adults (18–50 years old) increasing their alcohol consumption during lockdown (Neill et al. 2020; Pollard et al. 2020; Rolland et al. 2020; Sanchez et al. 2020; Vanderbruggen et al. 2020; Capasso et al. 2021; Killgore et al. 2021). For instance, a Polish study observed that differences in drinking frequencies between age groups also depended on the type of alcoholic beverage consumed (Szajnoga et al. 2021). It noted no significant results concerning age and consumption of beer and spirits. However, a significant relationship was found between age and wine consumption, with the highest consumption among those aged 18–24, and decreasing with age. This supports findings that younger age categories consumed more alcohol during the COVID‐19 pandemic (Szajnoga et al. 2021). Moreover, another study reported overall changes in consumption among younger people, such as higher frequency of drinking but decreased number of drinks per drinking occasion (Graupensperger et al. 2021). Conversely, other studies reported a decrease in alcohol consumption among younger adults (Hendriksen et al. 2022; Merlo, Hendriksen, et al. 2021; Sallie et al. 2020; Schmits and Glowacz 2022; Karadayian et al. 2023). A specific subset of younger adults—namely students—have generally displayed a decrease in alcohol consumption during lockdown (see Section 3.3.2.).

#### 3.1.3 Race

Limited information is available concerning the relationship between race and alcohol consumption during the pandemic. The retrieved studies on interracial differences in alcohol consumption changes focused on the number of days consuming alcohol, total number of drinks consumed, and binge drinking during the pandemic. A few studies reported higher consumption rates among non‐white individuals (Barbosa et al. 2021; Salerno et al. 2021; Acuff et al. 2022; Jaguga et al. 2022). Conversely, three studies reported increases in binge drinking and frequent drinking among white ethnic groups (Pollard et al. 2020; Wardell et al. 2020; Charles et al. 2021). Clear conclusions regarding the relationship between different races and alcohol consumption throughout the pandemic are hard to make considering the limited information available.

### 3.2 Socioeconomic Status

#### 3.2.1 Employment Status

A study conducted in the USA found increased alcohol consumption among a third of both employed and unemployed respondents (Capasso et al. 2021). A Belgian study noted those being unemployed to have a 36% greater chance of drinking more alcohol compared to those working from home (Vanderbruggen et al. 2020). Similarly, two other studies reported increased alcohol use related to job loss (Rossinot et al. 2020; Killgore et al. 2021). Other studies reported increased alcohol use among those working from home (Panagiotidis et al. 2020; Schmits and Glowacz 2022). A Polish study reported a greater increase in alcohol consumption among participants whose work pattern was impacted (e.g., 22.5% of those working from home, 26% of those temporarily laid‐off), whereas among the unemployed, less frequent alcohol use was observed (41.5%) (Szajnoga et al. 2021).

On the contrary, unemployment might also have a protective effect, as a decrease in alcohol consumption has been observed (Szajnoga et al. 2021; Valente et al. 2021). Moreover, a study conducted in France showed that lack of work was a protective factor for alcohol consumption. However, financial worries associated with unemployment might be a stronger predictor of increased alcohol consumption, suggesting that financial concerns are more likely to lead to higher consumption than the lack of employment itself (Rossinot et al. 2020). Nevertheless, other research has shown no relationship between employment status and alcohol consumption (Sidor and Rzymski 2020; Bramness et al. 2021).

Taken together, both work and being unemployed have been reported to either protective or risk factors in terms of alcohol consumption. On the one hand, work gives meaning to life and satisfaction which theoretically does not impact drinking behavior. Alternatively, work‐related stress can be associated with increased alcohol consumption. Similarly, unemployment can result in negative mood and reduced quality of life which may result in increased alcohol consumption as a coping mechanism. On the other hand, being unemployed lacks the impact of work‐related stress, and in combination with lockdowns, this may have resulted in less alcohol consumption.

#### 3.2.2 Working in Healthcare

In relation to employment, healthcare‐related professions were explored due to the sector's association with stressful working conditions and exposure to the coronavirus. However, results were limited and contradictory. A couple of studies mention a decrease in alcohol consumption among healthcare workers (Vanderbruggen et al. 2020; Oksanen et al. 2021). However, other studies reported increased alcohol consumption among healthcare workers (Sallie et al. 2020; Wozniak et al. 2021; Jaguga et al. 2022). A Belgian study (Vanderbruggen et al. 2020) found that non‐healthcare professionals were more prone to increase their drinking, with a 40% greater chance of consuming more alcohol compared to healthcare professionals. Other studies found no statistical difference in daily alcohol use among healthcare workers and other professionals (Mongeau‐Pérusse et al. 2021; Oksanen et al. 2021).

#### 3.2.3 Income

Regarding income, mixed results have been found. A study conducted in Belgium examining whether loss of income played a pivotal role in altered alcohol consumption during the pandemic did not find significant results (Schmits and Glowacz 2022). An Australian study, however, reported that higher income was correlated with increased alcohol use (Neill et al. 2020), and studies conducted in Latin America and the UK also showed a positive relationship, with higher incomes correlating with increased alcohol consumption (Garcia‐Cerde et al. 2021; Garnett et al. 2021; Valente et al. 2021). Conversely, a cross‐sectional study conducted in Europe observed that individuals with low or average income were more likely to report an increase in alcohol consumption, while high‐income respondents displayed the biggest reduction in alcohol use during the pandemic (Kilian et al. 2021).

### 3.3 Education

#### 3.3.1 Difference in Educational Level

Most studies examining the effect of educational level on alcohol consumption indicate those with higher education are more susceptible to increased alcohol consumption during the COVID‐19 pandemic (Rolland et al. 2020; Zajacova et al. 2020; Capasso et al. 2021; Bloomfield et al. 2022; Coulaud et al. 2022; Schmits and Glowacz 2022; Sumetsky et al. 2023). A study conducted in the U.S. for instance, found greater increases in alcohol consumption (32% of the study sample) in those with higher education levels compared to those without a bachelor's degree (26% of the study sample) (Capasso et al. 2021). Interestingly, a Belgian study investigating the effects of educational background on alcohol consumption found no significant correlations between education level and alcohol consumption (Vanderbruggen et al. 2020). Similarly, Sidor and Rzymski (2020) reported no significant differences in alcohol consumption according to educational level. However, two other studies showed that individuals with a lower level of education were more at risk of increasing their alcohol consumption (Koopmann et al. 2020; Salerno et al. 2021).

#### 3.3.2 Students

Research on the effects of the pandemic on alcohol consumption amongst students found that, in general, students decreased their alcohol consumption during the COVID‐19 pandemic, especially among those moving to live with their parents (Clare et al. 2021). However, it is important to note that the decrease in alcohol use refers to the quantity ingested rather than the frequency of use, which on average has increased (Dumas et al. 2020; H. R. White et al. 2020; Evans et al. 2021; Jaffe et al. 2021; Merlo, Hendriksen, et al. 2021; Rogés et al. 2021; Ryerson et al. 2021; Valente et al. 2021; Schmits and Glowacz 2022). One study among U.S. college students found that while drinking quantity decreased, drinking frequency increased. In this study, it was observed that students drank one more day in a typical week, but smaller quantities of alcohol were consumed per sitting (H. R. White et al. 2020). Similarly, other studies have supported this increase in frequency but reduction in overall quantity consumed per occasion (Vanderbruggen et al. 2020; Graupensperger et al. 2021).

Conversely, another study in students reported higher alcohol consumption during the COVID‐19 pandemic (Charles et al. 2021). A study conducted in Spain among university students founds that living together with other students was a risk factor for higher levels of alcohol use (Delgado‐Lobete et al. 2020). Furthermore, a study among Slovakian students examined the relationship between sex and income characteristics in relation to alcohol consumption and reported a significant relationship between being male and having a higher incomes with harmful alcohol use. In contrast, across all income groups females had a low‐risk of harmful alcohol use (Gavurova et al. 2020).

### 3.4 Living Situation

#### 3.4.1 Relationship Status

Only few studies examined the impact of relationship status on alcohol consumption during the COVID‐19 pandemic. A Greek study found no differences in alcohol consumption between those married and unmarried after the enforcement of COVID‐19 measures (Panagiotidis et al. 2020), while a Polish study reported that those in a relationship consumed significantly more alcohol compared to singles (Chodkiewicz et al. 2020).

#### 3.4.2 Children at Home

One consequence of the COVID‐19 pandemic was the closure of establishments such as schools and the introduction of at‐home childcare and homeschooling. Studies investigating the effect of having children at home during the COVID‐19 pandemic on alcohol consumption generally reached a consensus: those with children in their household and caregiving responsibilities were more likely to increase their alcohol consumption (Boschuetz et al. 2020; Chodkiewicz et al. 2020; Ingram et al. 2020; Rodriguez et al. 2020; Sallie et al. 2020; Vanderbruggen et al. 2020; Acuff et al. 2022; MacMillan et al. 2022; Schmits and Glowacz 2022; Colton et al. 2023). A Belgian study noted that for every extra child found in the family, the chance of increasing one's alcohol consumption increased by 22% (Vanderbruggen et al. 2020).

However, a U.S. study examining heavy episodic drinking found that drinking decreased among households with children compared to those without (Valente et al. 2021).

#### 3.4.3 Place of Residence

The potential impact of place of residence, that is, urban versus countryside, on alcohol consumption during the COVID‐19 pandemic has also been investigated. A US study found slight increases in alcohol consumption among those living in suburban and urban areas (31%, *n* = 5850), compared those living in rural areas (25%) (Capasso et al. 2021). This was further corroborated by a Polish study, which observed that those living in rural areas reported less frequent alcohol consumption, whereas those living in larger cities reported increased alcohol consumption during the COVID‐19 pandemic (Szajnoga et al. 2021). Moreover, this study observed the frequency of alcohol intake to be positively correlated to city size. Therefore, the larger the city, the higher the percentage of individuals increasing their alcohol intake (Szajnoga et al. 2021). In contrast, a Belgian study found no significant relationship between living in a city and alcohol consumption during the COVID‐19 pandemic (Vanderbruggen et al. 2020). In line, a Polish study also did not find significant effects of living location on alcohol consumption (Pollard et al. 2020), whereas a Danish study showed only minor differences (Bloomfield et al. 2022).

#### 3.4.4 Social Support

An American study found that lack of social support has a significant negative effect on alcohol consumption; that is, those who reported having increased social support drank significantly less alcohol during the COVID‐19 pandemic (Lechner et al. 2020). However, the same study also found that having social support did not change how psychological distress led to increased alcohol use over time. This was true for both the amount of alcohol people drank and how often they drank (Lechner et al. 2020). In contrast, Whittaker and Kingston (2022) reported that the connection between COVID‐19 pandemic‐related stress and alcohol consumption did not seem to be influenced by social support. Nevertheless, the positive correlation between loneliness and the frequency of alcohol use per month suggests that people who were more lonely during the COVID‐19 pandemic consumed more alcohol (Whittaker and Kingston 2022).

#### 3.4.5 Nearby Presence and Perceived Risk of COVID‐19

Individuals residing in states with elevated COVID‐19 burden initially reported a greater average number of days of drinking at the onset of the epidemic (McKetta et al. 2021). However, there was no indication of additional increases in alcohol consumption as the epidemic advanced. Fear and worry related to contracting COVID‐19 were correlated with increased alcohol use (Garnett et al. 2021; Taylor et al. 2021; Wozniak et al. 2021). Respondents in states with lower COVID‐19 burdens also exhibited a rise in the number of drinking days throughout the initial phase of the pandemic (McKetta et al. 2021). Conversely, Individuals who believed they faced a heightened risk of contracting COVID‐19 or anticipated a more severe impact from the coronavirus disease were slightly less inclined to escalate their alcohol consumption compared to those who perceived a lower risk and anticipated lower severity (Capasso et al. 2021).

### 3.5 Mental Health

#### 3.5.1 Mental Health

Of concern is the number of people claiming to have increased their alcohol consumption as a result of poor mental health during the COVID‐19 pandemic. Mental health encompasses emotions, psychological states, and social well‐being. While each article considered here does not necessarily measure mental health in the same way and with the same scales, there is a strong consensus that poor mental health is strongly related to greater alcohol consumption. These observations were made across a variety of samples, including people of different nationalities, ages, genders, occupational statuses, university students. One study in 1491 Australian adults reported almost 30% of participants to be using alcohol as a coping mechanism for increased psychological distress, thereby increasing their consumption (Stanton et al. 2020). An American study reported similar findings, with 60% stating they increased their alcohol consumption compared to pre‐COVID‐19, and of these, 46% attributed the increase in their drinking to heightened stress (Grossman et al. 2020). Another US study reported that of the 29% of the sample (*n* = 12,910) who claimed to have increased their alcohol consumption following the lockdown period, 59% experienced feelings of anxiety and 41% feelings of depression (Capasso et al. 2021). Overall, several studies mentioned the negative role of stress, depression, anxiety, and loneliness on drinking patterns, leading specifically to increased alcohol use (Gavurova et al. 2020; Koopmann et al. 2020; Merlo, Severeijns, et al. 2021a; Moura et al. 2023). Nevertheless, a few other studies found no significant relationship between stress and anxiety, and increased alcohol consumption (Mongeau‐Pérusse et al. 2021; Bloomfield et al. 2022).

#### 3.5.2 Binge Drinking

Different findings have been reported for the number of people binge drinking following the onset of the COVID‐19 pandemic. A Norwegian study reported frequent binge drinking among 14% of their sample, with the largest proportion among younger respondents (30%) (Alpers et al. 2021). Similarly increased binge drinking has been observed by a few other studies, corroborating the risk of binge drinking during lockdown periods (McPhee et al. 2020; Silczuk 2020; Wardell et al. 2020; Weerakoon et al. 2021). However, other studies reported a decrease in binge dinking. For example, a U.S. study found binge drinking patterns to have decreased, from 45% to 29.7% during the COVID‐19 pandemic (*n* = 417) (Boschuetz et al. 2020). Other studies also found a decrease in binge drinking among individuals (Ammar et al. 2020; Dumas et al. 2020), or no significant changes from pre‐COVID‐19 drinking (Grossman et al. 2020). Given the potential damage binge drinking can cause, it is important to look at factors and characteristics that may trigger increases or decreases in binge drinking habits. For example, one of the aforementioned studies found that non‐white participants engaged significantly more often in binge drinking activities during the COVID‐19 pandemic compared to white participants (14% vs. 6%) (Grossman et al. 2020). Furthermore, another study found sex to be a significant factor for binge drinking during the pandemic, with females reporting binge drinking more frequently than males (Boschuetz et al. 2020; Silczuk 2020; Jaguga et al. 2022). This contrasts with the Norwegian study which noted that males were up to three times more likely to engage in binge drinking behavior [103]. Weerakoon et al. (2021) reported that, amongst those already binge drinking, after considering factors like age and income, the likelihood of binge drinking increased by 1.19 times for each additional week spent at home during the COVID‐19 pandemic. Lastly, the odds of engaging in binge drinking behavior also increased among those experiencing depressive symptoms, those previously diagnosed with depression, and those with children (Boschuetz et al. 2020; Gritsenko et al. 2021).

## 4 Discussion

The COVID‐19 pandemic has exerted diverse effects on individuals' lives, ranging from isolation and diminished social interactions to remote work, job losses, alterations in income and financial circumstances, campus closures for students, and home‐schooling for parents. Daily routines underwent significant changes, resulting in both healthier and less healthy habits. Of particular concern and interest among these unhealthy behaviors is the heightened alcohol consumption, given its established association with poor mental health and its known impact on the immune system, elevating susceptibility for developing chronic diseases (Ammar et al. 2020; Brooks et al. 2020; Fitzgerald et al. 2020; Son et al. 2020; Catucci et al. 2021; Bühlmeier et al. 2022). Thus, achieving a greater understanding of the impact and effect of the factors explored in this review can help identify populations at higher risk of engaging in these behaviors and develop adequate support and preventative solutions to address the harmful consequence of heavy alcohol use.

Overall, this review showed that across countries, both increases and decreases of alcohol consumption have been observed. Although most respondents claim not changing their drinking habits, around 23% reported drinking less alcohol during the pandemic and 26% have reported drinking more alcohol during the pandemic. Thus, it is important to understand the underlying factors at play when it comes to changes in drinking behavior.

With regards to demographic factors, sex, age, and race were evaluated. A few studies found no differences in alcohol use between males and females during the pandemic, implying motives for drinking, such as boredom, conviviality, and loneliness, may not have influenced males and females differently (Chodkiewicz et al. 2020; Grossman et al. 2020; Gritsenko et al. 2021; Kilian et al. 2021; Szajnoga et al. 2021). However, an abundance of studies suggests that females might be more vulnerable to increases in alcohol consumption and high‐risk behaviors during social distancing (Boschuetz et al. 2020; Pollard et al. 2020; Rodriguez et al. 2020; Barbosa et al. 2021; Silczuk 2020; Steffen et al. 2021; Capasso et al. 2021; Salerno et al. 2021; Wozniak et al. 2021; Acuff et al. 2022; Jaguga et al. 2022). It is hypothesized that females are generally more likely to be involved with childcare (Sallie et al. 2020), and therefore the new circumstances of having to home‐school following the lockdown, while possibly having to simultaneously work from home, may cause extra stress and pressure. This added stress and pressure may be another explanation as to why females may consumed more alcohol during the lockdown periods compared to before the COVID‐19 pandemic (Silczuk 2020). Fewer articles reported males increasing their alcohol consumption, potentially linked to financial factors, such as rising unemployment rates, decreases in income, and pressure to financially support their family (Gritsenko et al. 2021; Killgore et al. 2021).

A discernible pattern appeared indicating a rise in alcohol consumption among young to middle‐aged adults (18–50 years old) during lockdown (Neill et al. 2020; Pollard et al. 2020; Rolland et al. 2020; Sanchez et al. 2020; Vanderbruggen et al. 2020; Capasso et al. 2021; Killgore et al. 2021). However, it is important to emphasize that the definition of age groups and measures of drinking patterns differ across these studies, thus different results could emerge if analyzed differently. A few studies have also observed that younger age groups are more likely to change their consumption overall, whether it is upwards or downwards (Biddle et al. 2020; Zajacova et al. 2020; Evans et al. 2021). Specifically, greater alcohol use was associated with financial worries and psychological states, such as depression, stress, and anxiety caused by unpredictability of the pandemic (Capasso et al. 2021; Niedzwiedz et al. 2021).

On the contrary, only a couple of articles reported decreased alcohol consumption and frequency among the younger age groups, mainly linked to physical distancing, closing of restaurants and bars, and canceled events, thus reducing social drinking opportunities (Callinan et al. 2021; Steffen et al. 2021). While these restrictions do impact drinking opportunities, it appeared that particular the younger age groups arranged other alternatives (e.g., drinking together at home, or organizing private parties at home) to continue their social activities, including alcohol consumption.

In examining interracial variations in alcohol consumption changes during the pandemic, differences were found in the number of days of alcohol consumption, the total number of drinks consumed, and instances of binge drinking. Some studies indicated higher consumption rates during the pandemic among non‐white individuals (Barbosa et al. 2021; Salerno et al. 2021; Acuff et al. 2022; Jaguga et al. 2022). Conversely, three other studies reported an increase in binge drinking and frequent drinking, specifically among white ethnic groups (Pollard et al. 2020; Wardell et al. 2020; Charles et al. 2021). Additional research must be done to correctly identify whether race has been a factor of influence on alcohol consumption throughout this pandemic, as this may highlight disproportionate impacts of the pandemic on different populations. A study that examined the difference in alcohol drinking patterns between Black and White individuals pre‐pandemic showed that White participants were the most likely to drink, while Black participants had the highest overall alcohol intake and frequency of heavy drinking. Moreover, Hispanics and Native Americans drank less frequently than European‐origin whites but consumed more alcohol on drinking days. These findings confirm the strong impact of cultural influences on drinking behavior (Dawson 1998).

Secondly, socio‐economic variables were reviewed, including employment status, working in the healthcare sector, income, education level and being a student. Varied results are observed regarding employment status throughout the pandemic, suggesting changes to working status impacted drinking patterns. Specifically, job loss was seen to be a potential risk factor for greater alcohol use (Rossinot et al. 2020; Killgore et al. 2021), as was working from home (McPhee et al. 2020; Panagiotidis et al. 2020; Szajnoga et al. 2021). On the contrary, unemployment might have a protective effect, as a decrease in alcohol consumption has been observed (Panagiotidis et al. 2020; Valente et al. 2021). The extensive disruptions to daily life caused by the pandemic and their lasting effects are significant as societal and economic upheavals can greatly influence public health and well‐being. Furthermore, they can lead to negative health behaviors such as greater consumption of alcohol, especially as individuals contend with job insecurity, changes in their daily routines, increased feelings of uncertainty, and may use drinking as a coping mechanism during hardships.

Furthermore, with regards to employment sector, individuals working in healthcare were investigated, as they played an essential role in helping patients suffering from the coronavirus. Again, some studies mention a decrease in alcohol consumption, whereas others reported an increase of alcohol consumption among healthcare workers (Sallie et al. 2020; Vanderbruggen et al. 2020; Mongeau‐Pérusse et al. 2021; Oksanen et al. 2021; Wozniak et al. 2021). Those working in healthcare have experienced exceptionally high workloads and work‐related pressure and distress in the recent years. Thus, there is potential for them to develop maladaptive coping mechanism in response to the high levels of workload, stress, depression, and anxiety associated with the pandemic. Furthermore, if those working in healthcare have other added responsibilities, such as children at home, perhaps needing to be home‐schooled, pressure and stress may increase even more, potentially augmenting their alcohol usage (Brooks et al. 2020; Callinan et al. 2021; Jacob et al. 2021).

When examining income, varied outcomes were noted; nevertheless, it seems that higher income might be linked to increased alcohol consumption. One study found no impact (Schmits and Glowacz 2022), while others indicated that higher incomes were associated with elevated alcohol consumption during the pandemic (Neill et al. 2020; Garcia‐Cerde et al. 2021; Garnett et al. 2021; Valente et al. 2021). The relationship is more evident concerning educational attainment, as it suggests that higher education is connected to a higher probability of alcohol use (Yan et al. 2020; Capasso et al. 2021; Bloomfield et al. 2022; Coulaud et al. 2022; Schmits and Glowacz 2022; Sumetsky et al. 2023). Interestingly, when looking at student samples specifically, an overall decrease in alcohol consumption was reported (H. R. White et al. 2020; Evans et al. 2021; Jaffe et al. 2021; Rogés et al. 2021; Ryerson et al. 2021; Valente et al. 2021; Acuff et al. 2022; Schmits and Glowacz 2022). Fewer social opportunities because of the closure of campuses and alcohol‐selling premises, as well as moving back home with parents, can partially explain this change. More specifically, additional studies have substantiated the rise in frequency while indicating a decrease in the overall quantity consumed per occasion (Graupensperger et al. 2021; Jackson et al. 2021).

More research is needed on the impact of relationship status and having children at home. Those having children in their household were generally more likely to increase their alcohol consumption. One consequence of the pandemic included the fact that more children had to stay at home and potentially be home‐schooled. For parents working full‐/part‐time, this added task can be perceived as a burden, potentially increasing stress levels (Boschuetz et al. 2020; Ingram et al. 2020; Sallie et al. 2020; Vanderbruggen et al. 2020; Acuff et al. 2022; MacMillan et al. 2022; Schmits and Glowacz 2022).

Regarding place of residence, mixed results were found with some studies attributing higher alcohol intake to living in cities (Capasso et al. 2021; Szajnoga et al. 2021) and another study reporting no difference for place of residence (Vanderbruggen et al. 2020).

Social support can create a safe haven, an environment in which one feels comfortable and cared for, something that may be especially important during a time in which people have been advised to physically isolate, and social gatherings have been limited. Two studies found that lack of social support has a significant negative effect on alcohol consumption, leading to higher alcohol use (Lechner et al. 2020; Gray et al. 2023). On the other hand, Whittaker and Kingston (2022) reported that the connection between pandemic‐related stress and alcohol consumption did not seem to be influenced by social support, although a positive relationship between loneliness and the frequency of alcohol use per month suggests that substance use may fluctuate based on this factor. Earlier research has shown that lack of social support can contribute to feelings of loneliness, an emotion known to impact mental health (Kypri and McCambridge 2018). Moreover, the literature on loneliness and social isolation reveals that alcohol consumption may increase under such circumstances, as people engage in drinking behavior as a coping mechanism to manage the psychological distress they experience (Jane‐Llopis and Matytsina 2006; Courtney and Polich 2009). The latter may explain why social support and loneliness play an important role on drinking patters, and explain the fact that young adults moving back to live with their parents often had a protective effect. Moreover, it has been stated that social support can increase one's ability to cope with stress. As augmented stress levels have also been correlated with increased alcohol consumption, perhaps this is another reason why increased social support may moderate alcohol consumption (Kypri and McCambridge 2018).

The perceived risk of contracting COVID‐19 also impacted alcohol consumption during the COVID‐19 pandemic. There are studies that show a positive correlation between distress, perceived risk, and altered alcohol patterns (Brooks et al. 2020; Jacob et al. 2021; Schmits and Glowacz 2022). Perhaps stress arising from the pandemic triggers feelings of increased perceived risk, leading to alcohol usage as a coping mechanism (Garnett et al. 2021; Wozniak et al. 2021). Alternatively, Capasso et al. (2021) reported a protective effect of risk of contracting COVID‐19, as individuals who believed they faced a heightened risk or anticipated more severe consequences from COVID‐19 were less inclined to increase their alcohol consumption compared to those perceiving lower risk and severity. It is possible that public health communications highlighting the detrimental impact of alcohol on immune function could have acted as a deterrent to drinking among those expressing greater COVID‐19 concerns.

Next, studies reported an association of mental health (i.e., rates of stress, depression, anxiety, etc.) with increased alcohol consumption during the pandemic. These observations were made across countries and individuals of different ages, genders, socio‐economic backgrounds, and occupational statuses (Avery et al. 2020; Chodkiewicz et al. 2020; Gavurova et al. 2020; Koopmann et al. 2020; Moura et al. 2023). These studies raise an important concern about the impact of lockdowns on individuals' mental status and related drinking behavior as a coping mechanism.

Lastly, binge drinking patterns have been impacted by the COVID‐19 pandemic. According to the NIAAA (2023), binge drinking is the consumption of alcohol leading to a blood alcohol concentration of 0.08% or higher. Binge drinking is an important health risk and must not be underestimated. For example, binge drinking has been associated with drunk driving, alcohol poisoning, unintentional injuries, and other socio‐economic costs. Furthermore, binge drinking has been linked to long‐term alcohol use disorders as well as dependence, which may not only lead to diseases such as liver disease or cancer but also impair the functioning of the immune system. The latter is of significant relevance during the COVID‐19 pandemic, as poorer immune fitness has been shown to be the most important predictor of the number and severity of COVID‐19 symptoms (Kiani et al. 2022). Other studies found that individuals who consumed alcohol faced an elevated risk of experiencing symptomatic COVID‐19, and those engaging in excessive drinking were particularly susceptible to COVID‐19 hospitalization (Wei et al. 2023). Thus, alcohol consumption exacerbates the severity of COVID‐19 and worsens clinical outcomes. Some factors, such as sex and race may influence binge drinking patterns (Boschuetz et al. 2020; Alpers et al. 2021; Gritsenko et al. 2021). It is important to further evaluate which factors may increase the risk of binge drinking so that preventative measures can be taken.

### 4.1 Limitations

The findings should be viewed in the context of the limitations of the studies that were reviewed. Firstly, all data from these studies were collected at different times during the COVID‐19 pandemic, thus effects could differ based on when during the pandemic respondents completed the research. Secondly, the studies originate in different countries, where the type, duration, and severity of (lockdown) restrictions differed. In addition, cross‐cultural differences in drinking patterns add to the complexity of accurately assessing alcohol consumption (Bloomfield et al. 2003). In this context, although a variety of countries were included in this review, South America and Asia were underrepresented in comparison to Europe and North America. Thirdly, it could be argued that publication bias toward publishing studies with statistically significant outcomes may have influenced the published data on alcohol consumption during the COVID‐19 pandemic. However, given the many studies summarized in this review reported negative results (no change in alcohol consumption), it is unlikely that there has been a publication bias toward not publishing non‐significant findings.

Finally, potential recall bias must be acknowledged as studies included self‐report surveys, and participants had to report their alcohol consumption retrospectively. Moreover, studies included different methods and scales to measure changes in drinking behavior and mood. Thus, recall bias may have played a role and reduce the accuracy of results in comparison to longitudinal studies. On the one hand, it can be argued that lockdown is an exceptional period, and as such, individuals should be able to recall the impact relatively well. Alternatively, individuals may have exaggerated the lockdown effects or idealized the period prior to the COVID‐19 pandemic and associated lockdowns.

### 4.2 Suggestions for Future Research

In this review several factors were identified that either increased alcohol consumption or showed to be protective factors. For example, heightened stress during the COVID‐19 pandemic may have increased alcohol consumption, whereas sufficient social support from family and friends may have been a protective factor. Future research should further investigate these factors. Whereas some on these factors can not be modified (e.g., and demographics such as sex and age), other factors putting individuals at risk (e.g., mood, mental resilience, lifestyle, and level of social support) are modifiable risk factors for health, disease and wellbeing that also impact coping strategies, including alcohol consumption. These factors deserve more attention to prepare the general population for future pandemics, for example by informing them about more healthy coping strategies (e.g., preserving an adequate immune fitness by attaining a healthy diet and regular physical activity, or apply meditation to reduce or prevent stress). In addition, a critical review of the restrictions and lockdown measures during the COVID‐19 pandemic should be conducted. It should further be evaluated to what extent these measures positively contributed toward reducing the spread of the SARS‐CoV‐2 virus. This effect should then be weighed in relation to the possible negative effects of these restrictions on health and wellbeing of individuals.

## 5 Conclusions

Overall, the pandemic has contributed to changes in alcohol drinking pattens, with a significant proportion of individuals either increasing (26%) or decreasing (23%) their alcohol intake. Among those who consumed more alcohol during the pandemic, being female, having a child at home, higher income, higher educational level, and a decline in mental health are key factors that drove greater alcohol use. Thus, these populations should be considered carefully, and tailored solutions to minimise the impact of future pandemic‐like situations should be examined and implemented. On the other hand, it would be useful to learn more about and further understand the factors at play among those who reduced their drinking to potentially identify interventions and solutions.

Insights from prior studies on post‐disaster scenarios underscore the necessity of prompt intervention regarding substance use to alleviate the heightened mental health repercussions of the pandemic (Capasso et al. 2021). Public health infrastructures should proactively engage individuals with preexisting mental health disorders, including substance use disorders, whose conditions may be exacerbated by circumstances like the COVID‐19 pandemic.

As a result of the various factors identified to have an impact on drinking patterns during the pandemic, preventing an increase in alcohol consumption would require a multifaceted approach that addresses individual behaviors, societal influences, and systemic factors. Moreover, the findings of this review underscores the importance of customising public health communication regarding substance use across different demographics. Additionally, there is a pressing need to enhance prevention and treatment strategies for individuals who are prone to problematic alcohol consumption as a response to stress, and outreach initiatives should prioritise populations at higher risk of both COVID‐19 susceptibility and problem alcohol use, such as older adults and individuals with a history of mental health conditions. By combining these strategies and tailoring them to specific cultural and contextual factors, communities and policymakers can work toward preventing the increase in alcohol consumption and promoting healthier behaviors.

## Author Contributions

**A. Merlo:** conceptualization, writing – original draft, writing – review and editing. **P.A. Hendriksen:** conceptualization, writing – original draft, writing – review and editing. **N.R. Severeijns:** conceptualization, writing – review and editing. **J. Garssen:** conceptualization, writing – review and editing. **G. Bruce:** conceptualization, writing – review and editing. **J.C. Verster:** conceptualization, writing – review and editing.

## Conflicts of Interest

Over the past 3 years, J.V. has received research grants from Danone and Inbiose and has acted as a consultant/advisor for Eisai, KNMP, Med Solutions, Mozand, Red Bull, Sen‐Jam Pharmaceutical, and Toast!. J.G. is part‐time employee of Nutricia Research and received research grants from Nutricia research foundation, Top Institute Pharma, Top Institute Food and Nutrition, GSK, STW, NWO, Friesland Campina, CCC, Raak‐Pro, and EU. The other authors have no potential conflicts of interest to disclose.

## Data Availability

The data that support the findings of this study are available from the corresponding author upon reasonable request.
